# Effect of Body Mass Index in Patients With Cardiogenic Shock Requiring Microaxial Flow Pump

**DOI:** 10.1016/j.jacasi.2025.03.003

**Published:** 2025-03-24

**Authors:** Yuki Katagiri, Yutaro Kasai, Mamoru Miyazaki, Ken Kuroda, Yuichiro Hosoi, Kohei Ishikawa, Hiroki Bota, Yuki Ikeda, Yohei Sotomi, Kenichi Matsutani, Kazumasa Yamasaki, Tomoyuki Tani, Takashi Ueda, Seiji Yamazaki, Shigeru Saito, J-PVAD Investigators

**Affiliations:** a Department of Cardiology, Sapporo Higashi Tokushukai Hospital, Sapporo, Japan; b Department of Cardiovascular Medicine, Kitasato University School of Medicine, Sagamihara, Japan; c Department of Cardiovascular Medicine, Osaka University Graduate School of Medicine, Osaka, Japan; d Department of Cardiovascular Surgery, Sapporo Higashi Tokushukai Hospital, Sapporo, Japan; e Department of Cardiology, Shonan Kamakura General Hospital, Kamakura, Japan

**Keywords:** body mass index, cardiogenic shock, Impella microaxial flow pump, mechanical circulatory support, obesity

## Abstract

**Background:**

The impact of obesity on mortality in patients with cardiogenic shock (CS) requiring microaxial flow pumps (mAFP) remains undetermined.

**Objectives:**

This study investigated the effect of body mass index (BMI) on mortality in CS patients treated with mAFP.

**Methods:**

Data from 3,636 consecutive CS patients treated with Impella mAFP in the J-PVAD (Japanese Registry for Percutaneous Ventricular Assist Device) nationwide prospective registry in Japan between February 2020 and December 2022 were analyzed. Patients were stratified into 5 BMI categories: underweight (<18.5 kg/m^2^), normal weight (18.5-22.9 kg/m^2^), overweight (23.0-24.9 kg/m^2^), obesity (25.0-29.9 kg/m^2^), and severe obesity (≥30.0 kg/m^2^). Multivariate Cox regression analysis assessed the relationship between BMI and 30-day mortality.

**Results:**

Crude 30-day mortality increased incrementally with higher BMI categories. Adjusted HRs for 30-day mortality (normal weight as reference) were 0.71 (95% CI [CI]: 0.56-0.90; *P =* 0.005) for underweight, 1.03 (95% CI: 0.88-1.21; *P =* 0.681) for overweight, 1.37 (95% CI: 1.19-1.57; *P <* 0.001) for obesity, and 2.00 (95% CI: 1.66-2.41; *P <* 0.001) for severe obesity. Patients in the underweight and severe obesity groups experienced a higher incidence of bleeding after percutaneous coronary intervention under mAFP, whereas hemolysis increased with higher BMI categories. Bleeding and hemolysis were associated with mortality only in patients who were underweight.

**Conclusions:**

Higher BMI was associated with increased mortality in CS patients treated with mAFP. Although patients who were underweight demonstrated overall favorable survival outcomes, bleeding and hemolysis contributed to mortality in this group. Further research is needed to explore whether a BMI-based approach can improve clinical outcomes. (Japanese registry for Percutaneous Ventricular Assist Device; UMIN000033603)

The mortality rate among patients with cardiogenic shock (CS) remains high, with recent data indicating in-hospital mortality rates of approximately 35% to 45%, despite advances in therapeutic strategies.^1^ Mechanical circulatory support (MCS) has been used to manage patients with CS, although robust evidence from randomized controlled trials demonstrating efficacy was lacking.^2^^,^^3^ Most recently, the DanGer shock trial, which randomized patients with ST-segment elevation myocardial infarction and CS to either treatment with an Impella CP microaxial flow pump (mAFP) (Abiomed) plus standard care or standard care alone, reported improved survival in those receiving mAFP.^4^ This trial is regarded as a landmark, potentially increasing the global penetration of mAFP in treating CS.

Obesity is a substantial public health concern because of its established relationship with cardiovascular disease and adverse outcomes in the general population^5^^,^^6^; however, several studies have suggested that an increase in body mass index (BMI), a widely used measure of body mass relative to height for assessing obesity, is associated with improved prognosis in patients with heart failure^7^^,^^8^ or ischemic heart disease,^9^^,^^10^ showing either an inverse linear or U-shaped association between BMI and mortality, often referred to as the “obesity paradox.” In the largest study to date investigating the impact of BMI in patients with CS requiring MCS, obesity was associated with higher in-hospital mortality and increased bleeding complications; however, the majority of the study population consisted of those who received an intra-aortic balloon pump,^11^ limiting the generalizability to patients treated with mAFP. In patients treated with mAFP, bleeding and hemolysis remain important complications^12^; however, their impact on mortality has not been established, and the interaction with the degree of obesity warrants further exploration.

Thus, the effect of BMI on mortality and adverse events in patients with CS requiring mAFP has yet to be elucidated. Therefore, this study aimed to investigate the association between BMI and clinical outcomes in patients with CS receiving hemodynamic support by mAFP, utilizing data from a Japanese nationwide database.

## Methods

### Data source

The J-PVAD (Japanese Registry for Percutaneous Ventricular Assist Device) is a nationwide, prospective registry that includes all consecutive patients treated with mAFP in Japan. The registry is led by the Impella committee of the Council for Clinical Use of Ventricular Assist Device Related Academic Societies, consisting of 10 academic organizations, to safely and effectively disseminate the use of mAFP devices.^13^ The J-PVAD collects data, including patient demographics, laboratory results, hemodynamic and device parameters, concomitant treatments, and adverse events such as survival status and device malfunction, all reported by investigators from participating institutions. Central monitoring was performed for the present J-PVAD data set. The study adhered to the principles of the Declaration of Helsinki, received approval from the Osaka University Ethics Committee (approval number: 17232, approval date: November 21, 2017), and was registered in the University hospital Medical Information Network (UMIN000033603). Written informed consent was waived because of complete data anonymization and the observational nature of the study. Data that support the study findings may be available upon permission from the Impella committee of the Council for Clinical Use of Ventricular Assist Device Related Academic Societies, through the corresponding author.

### Study population

**Figure 1:**
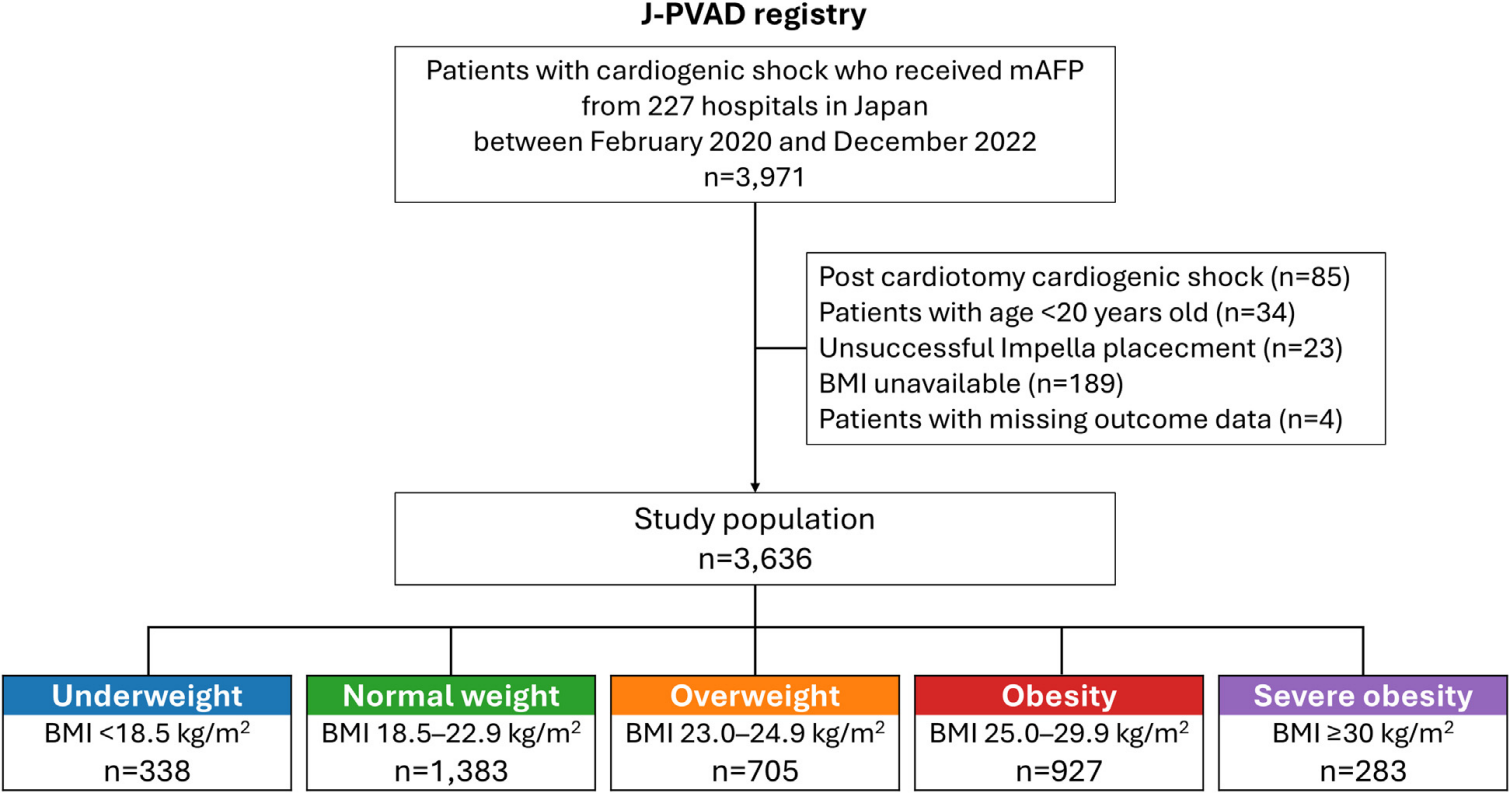
Study Flowchart BMI = body mass index; J-PAVD = Japanese Registry for Percutaneous Ventricular Assist Device; mAFP = microaxial flow pump.

This study analyzed patients with CS treated with mAFP between February 2020 and December 2022, using data from the J-PVAD registry. Exclusion criteria included patients younger than 20 years, and those with postcardiotomy CS, unsuccessful mAFP delivery, or missing BMI or outcome data. Of 3,971 patients with CS recorded in the registry, 3,636 were included in the present analysis (Figure 1).

### Microaxial flow pump device

During the study period, Impella 2.5, CP, 5.0, and 5.5 were available for patients with drug-resistant acute heart failure, including CS. Impella 2.5, CP, 5.0, and 5.5 are 12-, 14-, 21-, and 21-F–sized microaxial pumps.^14^ They were inserted either percutaneously via the femoral artery or surgically through exposure of the axillary or subclavian arteries with insertion via an artificial vessel, especially for Impella 5.0 and 5.5. Once positioned in the left ventricle, these pumps provided temporary circulatory support, delivering continuous blood flow up to 2.5, 3.7, 5.0, and 5.5 L/min, respectively. In Japan, Impella 2.5 and 5.0 were approved by the Pharmaceuticals and Medical Devices Agency of the Japanese Ministry of Health, Labor, and Welfare in September 2016, Impella CP in March 2019, and Impella 5.5 in November 2021 for the treatment of drug-resistant acute heart failure.

### Definitions and endpoints

BMI (kg/m^2^) was defined as the body weight (kg) divided by the square of the body height (m). Patients were categorized into BMI groups based on Asian cutoffs proposed by the World Health Organization as follows: underweight (<18.5 kg/m^2^), normal weight (18.5-22.9 kg/m^2^), overweight (23.0-24.9 kg/m^2^), obesity (25.0-29.9 kg/m^2^), and severe obesity (≥30.0 kg/m^2^).^15^

CS was defined as a shock state caused by cardiac disease meeting at least 1 major criterion and any minor criterion. The major criteria included the following: 1) systolic blood pressure <100 mm Hg with a heart rate <60 or ≥100 beats/min; and 2) a systolic blood pressure decrease >30 mm Hg from usual values. Minor criteria included signs of peripheral circulatory failure, such as cold sweat, skin pallor, cyanosis, capillary refill time ≥2 seconds, or consciousness disturbance. A condition requiring the use of intravenous inotropes and/or MCS (eg, extracorporeal membrane oxygenation [ECMO] or intra-aortic balloon pump) to maintain the blood pressure and cardiac index was also regarded as CS.

The primary endpoint of the study was 30-day all-cause mortality. Secondary endpoints were the incidence of complications, including bleeding, hemolysis, lower limb ischemia, access-related vascular injury, and cerebrovascular accident. Bleeding was defined as bleeding requiring transfusion. Hemolysis was defined by an increase of >40 mg/dL in plasma-free hemoglobin levels or a clinically significant increase in lactate dehydrogenase and indirect bilirubin levels along with a decrease in hemoglobin levels. Lower limb ischemia was defined as clinical evidence of lower limb hypoperfusion requiring intervention. Access-related vascular injuries were defined as conditions requiring diagnostic testing and therapeutic intervention, such as a pseudoaneurysm, arteriovenous fistula, vascular thrombosis, vascular dissection, perforation, rupture, or vascular stenosis related to mAFP devices. Cerebrovascular accidents were defined as clinically significant neurological deficits and imaging evidence of cerebral infarction or intracranial hemorrhage.

### Statistical analysis

Categorical variables are reported as numbers (percentages) and are compared using chi-square or Fisher exact test, as appropriate. Continuous variables were presented as median (IQR) and compared using the Kruskal-Wallis test. The Kaplan-Meier method was used to evaluate 30-day mortality, with the time of mAFP insertion treated as day 0, and the groups were compared using the log-rank test.

To assess the independent association between BMI and 30-day mortality, multivariate Cox regression analysis was performed, adjusting for the following prespecified baseline characteristics in addition to BMI: age, sex, etiology of CS, in-hospital cardiac arrest, use of ECMO, mean blood pressure, lactate, lactate dehydrogenase, total bilirubin, creatinine, and albumin. These variables were chosen based on a previous analysis in the J-PVAD data set.^16^ The proportional hazards assumption was verified visually by plotting scaled Schoenfeld residuals against time. The same multivariate model was used for subgroup analysis. Adjusted hazard ratio (aHR) in subgroups was color-mapped for comparison across subgroups. In addition, to investigate the associations of bleeding and hemolysis events with mortality, these variables were further included as time-updated binary covariates.^17^ For handling missing values in the multivariate analyses, random forest imputation was applied using the “missForest” package. All statistical analyses were performed using R software (version 4.4.1; R Foundation for Statistical Computing). Statistical significance was set at *P <* 0.05.

## Results

### Patient characteristics

**Figure 2:**
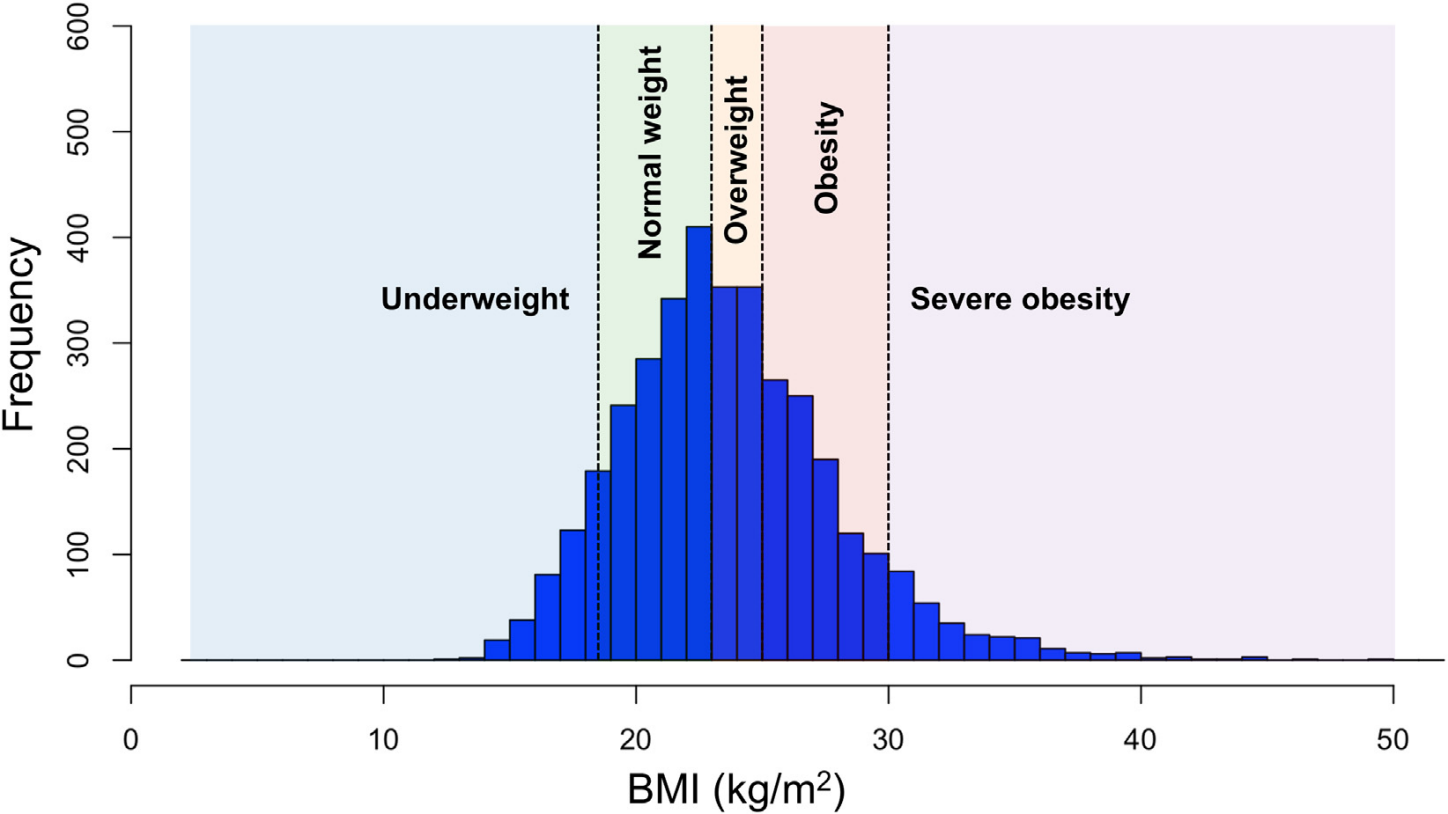
Histogram of BMI Distribution The body mass index (BMI) categories were defined as underweight (<18.5 kg/m^2^), normal weight (18.5-22.9 kg/m^2^), overweight (23.0-24.9 kg/m^2^), obesity (25.0-29.9 kg/m^2^), and severe obesity (≥30.0 kg/m^2^).^15^

Among the 3,636 patients included in the study, 338 (9.3%) were categorized as underweight, 1,383 (38.0%) as normal weight, 705 (19.4%) as overweight, 927 (25.5%) as obese, and 283 (7.8%) as severely obese (Figures 1 and 2). The baseline characteristics of the study population are presented in Table 1. Overall, the median age was 69.0 years (Q1-Q3: 58.0-77.0 years), with 809 (22.2%) women. Patients in higher BMI categories were younger, were more often men, and exhibited a larger body surface area. Regarding the indication for mAFP use, acute myocardial infarction (AMI) was the most common (n = 2,263 [62.2%]) across all groups, with the highest proportion observed in the overweight group. Myocarditis was most frequently observed in the underweight group. The prevalence of conventional atherosclerotic risk factors such as hypertension, dyslipidemia, diabetes, and smoking increased with higher BMI categories. Similarly, the frequency of cardiac arrest (both out-of-hospital and in-hospital), the performance of cardiopulmonary resuscitation, and ECMO use before mAFP implantation were more frequent in patients with higher BMI. Regarding laboratory values, albumin and creatinine levels were lowest in the underweight group. The missing numbers for baseline variables are summarized in Supplemental Table 1.

**Table 1:** Baseline Characteristics

	Overall (N = 3,636)	Underweight (n = 338)	Normal Weight (n = 1,383)	Overweight (n = 705)	Obesity (n = 927)	Severe Obesity (n = 283)	*P* Value
Age, y	69.0 (58.0-77.0)	73.0 (62.0-79.0)	71.0 (60.0-78.0)	71.0 (60.0-77.0)	65.0 (55.0-74.0)	61.0 (50.0-71.0)	<0.001
Female	809 (22.2)	118 (34.9)	341 (24.7)	128 (18.2)	161 (17.4)	61 (21.6)	<0.001
BMI, kg/m^2^	23.2 (20.8-26.1)	17.3 (16.4-18.0)	21.2 (20.0-22.1)	24.0 (23.5-24.5)	26.8 (25.9-27.9)	32.1 (30.8-34.6)	<0.001
BSA, m^2^	1.69 (1.56-1.83)	1.45 (1.36-1.54)	1.62 (1.50-1.71)	1.72 (1.62-1.81)	1.83 (1.73-1.93)	1.99 (1.85-2.11)	<0.001
Indication							<0.001
AMI	2,263 (62.2)	182 (53.8)	822 (59.4)	484 (68.7)	611 (65.9)	164 (58.0)	
Mechanical complications of MI	134 (3.7)	7 (2.1)	56 (4.0)	23 (3.3)	41 (4.4)	7 (2.5)	
Ischemic cardiomyopathy	245 (6.7)	31 (9.2)	100 (7.2)	42 (6.0)	52 (5.6)	20 (7.1)	
Myocarditis	336 (9.2)	40 (11.8)	151 (10.9)	52 (7.4)	73 (7.9)	20 (7.1)	
Nonischemic cardiomyopathy	198 (5.4)	26 (7.7)	78 (5.6)	28 (4.0)	42 (4.5)	24 (8.5)	
Valvular heart disease	93 (2.6)	15 (4.4)	43 (3.1)	17 (2.4)	12 (1.3)	6 (2.1)	
Ventricular arrhythmia	244 (6.7)	25 (7.4)	87 (6.3)	37 (5.2)	68 (7.3)	27 (9.5)	
Others	123 (3.4)	12 (3.6)	46 (3.3)	22 (3.1)	28 (3.0)	15 (5.3)	
Previous medical history							
Hypertension	2,103 (61.1)	177 (54.5)	748 (57.5)	415 (61.5)	577 (66.0)	186 (69.9)	<0.001
Dyslipidemia	1,524 (44.2)	126 (38.5)	517 (39.5)	309 (45.8)	447 (51.3)	125 (47.0)	<0.001
Diabetes	1,414 (40.6)	119 (36.4)	475 (36.0)	275 (40.4)	401 (45.1)	144 (53.5)	<0.001
Chronic kidney disease	1,082 (31.3)	116 (35.4)	391 (29.8)	223 (33.2)	269 (30.7)	83 (31.3)	0.268
Hemodialysis	179 (5.2)	23 (7.0)	65 (5.0)	34 (5.1)	44 (5.0)	13 (4.9)	0.650
Coronary artery disease	929 (26.9)	84 (26.1)	356 (27.0)	186 (27.5)	226 (25.7)	77 (29.3)	0.803
Myocardial infarction	685 (19.7)	53 (16.2)	248 (18.8)	141 (20.8)	184 (20.9)	59 (22.3)	0.220
Atrial fibrillation	329 (9.0)	36 (10.7)	133 (9.6)	56 (7.9)	76 (8.2)	28 (9.9)	0.448
Heart failure	844 (24.4)	104 (32.0)	317 (24.0)	157 (23.1)	185 (21.1)	81 (30.6)	<0.001
Ischemic stroke or TIA	274 (7.8)	24 (7.3)	113 (8.5)	54 (7.9)	66 (7.5)	17 (6.4)	0.765
Smoking	1,898 (61.4)	150 (51.2)	720 (60.1)	386 (64.0)	491 (64.0)	151 (65.9)	<0.001
Out-of-hospital cardiac arrest	830 (23.0)	44 (13.1)	288 (21.0)	173 (24.7)	239 (25.9)	86 (30.7)	<0.001
In-hospital cardiac arrest	1,212 (33.3)	99 (29.3)	428 (30.9)	238 (33.8)	329 (35.5)	118 (41.7)	0.002
Cardiopulmonary resuscitation	1,686 (46.4)	133 (39.3)	595 (43.0)	336 (47.7)	456 (49.2)	166 (58.7)	<0.001
Left ventricular ejection fraction, %	25.0 (20.0-35.0)	27.0 (20.0-35.0)	27.0 (20.0-35.0)	26.0 (20.0-40.0)	25.0 (20.0-35.0)	24.0 (15.0-31.5)	0.128
Inotropes	2,832 (77.9)	262 (77.5)	1,065 (77.0)	547 (77.6)	722 (77.9)	236 (83.4)	0.225
IABP before mAFP	341 (9.4)	32 (9.5)	126 (9.1)	55 (7.8)	91 (9.8)	37 (13.1)	0.139
ECMO before mAFP	1,155 (31.8)	82 (24.3)	397 (28.7)	222 (31.5)	319 (34.4)	135 (47.7)	<0.001
Vital signs at mAFP implantation							
Systolic blood pressure, mm Hg	87.0 (70.0-105.0)	84.0 (70.0-103.0)	87.0 (71.0-105.0)	87.0 (72.0-105.0)	86.0 (69.0-105.0)	85.0 (67.0-100.0)	0.114
Diastolic blood pressure, mm Hg	58.0 (42.0-72.0)	54.5 (40.0-70.0)	58.0 (42.0-72.0)	58.0 (44.0-71.0)	58.0 (43.0-71.0)	58.0 (43.0-74.0)	0.457
Mean blood pressure, mm Hg	67.0 (53.3-82.0)	65.3 (50.3-80.7)	67.0 (53.7-82.3)	67.7 (54.7-82.7)	66.7 (53.3-81.7)	66.7 (52.7-81.3)	0.468
Heart rate, beats/min	92.0 (71.0-110.5)	92.5 (72.0-110.0)	93.0 (73.0-111.0)	90.0 (69.0-110.0)	93.0 (70.0-112.0)	93.0 (72.0-110.0)	0.401
Laboratory values at mAFP implantation							
Lactate, mmol/L	5.00 (2.50-9.77)	4.78 (2.15-8.57)	4.97 (2.50-9.45)	5.00 (2.50-9.35)	5.13 (2.54-10.41)	5.66 (2.60-10.94)	0.088
Lactate dehydrogenase, IU/L	394.0 (260.0-737.0)	381.0 (263.0-644.0)	380.5 (259.0-701.0)	397.0 (258.0-795.0)	412.5 (257.0-761.0)	441.0 (281.0-854.0)	0.076
Total bilirubin, mg/dL	0.70 (0.50-1.09)	0.70 (0.50-1.10)	0.70 (0.50-1.00)	0.70 (0.46-1.10)	0.70 (0.50-1.01)	0.70 (0.41-1.30)	0.817
Albumin, g/dL	3.40 (2.90-3.80)	3.20 (2.70-3.60)	3.40 (2.90-3.80)	3.40 (2.90-3.90)	3.50 (2.90-3.90)	3.40 (2.80-3.80)	<0.001
Creatinine, mg/dL	1.24 (0.96-1.77)	1.17 (0.91-1.71)	1.21 (0.92-1.77)	1.25 (0.98-1.76)	1.26 (0.99-1.78)	1.32 (1.02-1.94)	0.002
PCI under mAFP	1,641 (45.1)	140 (41.4)	611 (44.2)	363 (51.5)	425 (45.8)	102 (36.0)	<0.001
mAFP device							0.005
Impella 2.5	134 (3.7)	15 (4.4)	63 (4.6)	32 (4.5)	21 (2.3)	3 (1.1)	
Impella CP	3,374 (92.8)	311 (92.0)	1,276 (92.3)	650 (92.2)	876 (94.5)	261 (92.2)	
Impella 5.0	98 (2.7)	7 (2.1)	35 (2.5)	18 (2.6)	22 (2.4)	16 (5.7)	
Impella 5.5	30 (0.8)	5 (1.5)	9 (0.7)	5 (0.7)	8 (0.9)	3 (1.1)	
Insertion site							0.014
Femoral	3,476 (95.6)	320 (94.7)	1,330 (96.2)	681 (96.6)	886 (95.6)	259 (91.5)	
Subclavian	149 (4.1)	18 (5.3)	49 (3.5)	22 (3.1)	36 (3.9)	24 (8.5)	
Others	11 (0.3)	0 (0.0)	4 (0.3)	2 (0.3)	5 (0.5)	0 (0.0)	

Values are median (Q1-Q3) or n (%).

AMI = acute myocardial infarction; BMI = body mass index; BSA = body surface area; ECMO = extracorporeal membrane oxygenation; IABP = intra-aortic balloon pump; mAFP = microaxial flow pump; MI = myocardial infarction; PCI = percutaneous coronary intervention; TIA = transient ischemic attack.

### BMI and mortality

**Figure 3:**
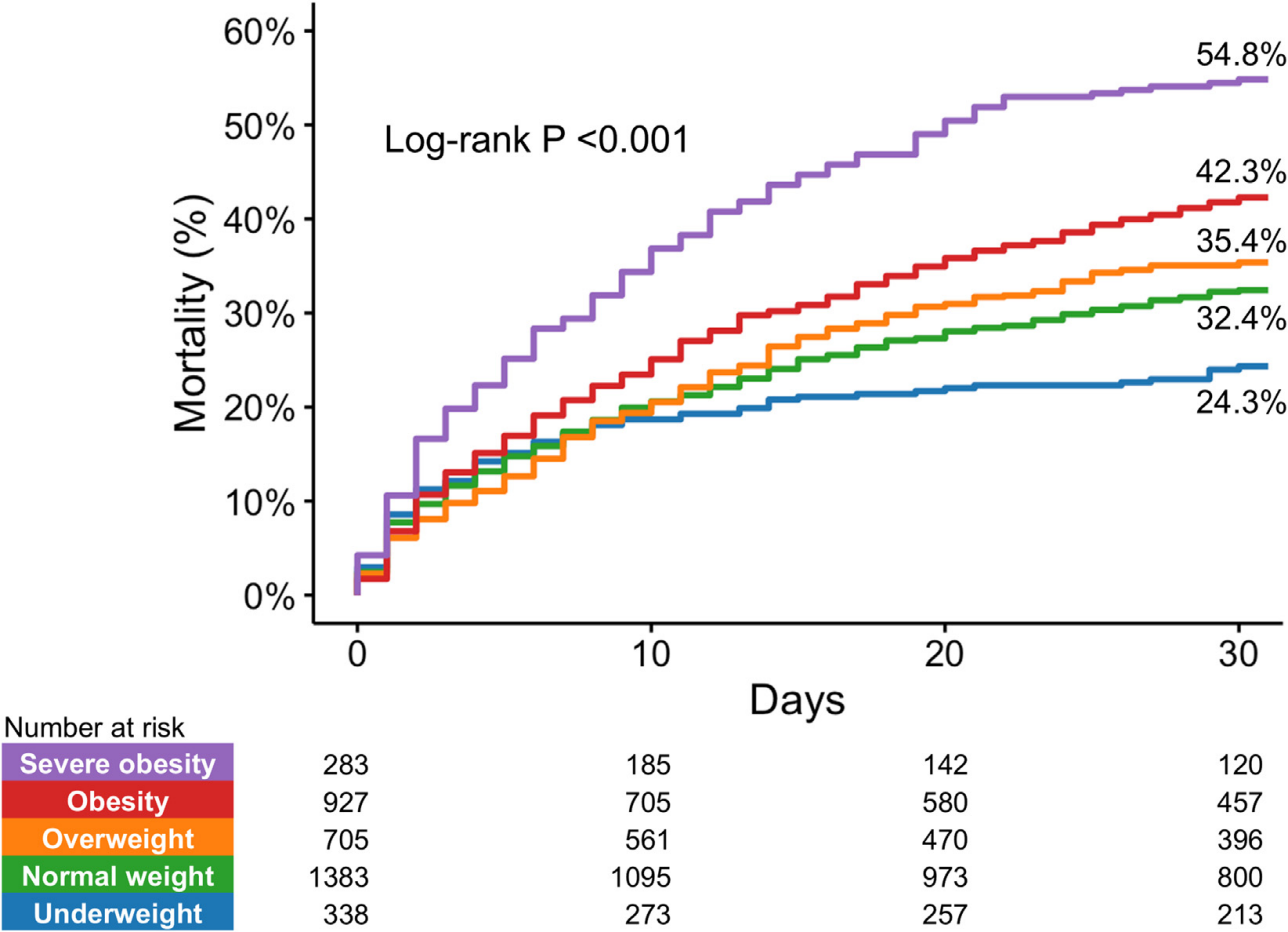
Kaplan-Meier Estimates of 30-Day Mortality According to Body Mass Index Categories The Kaplan-Meier curve demonstrated a significant increase in 30-day mortality with higher body mass index categories. Percentages indicate estimated mortality at 30 days after the first microaxial flow pump insertion.

Crude number (Table 2) and the Kaplan-Meier estimates of 30-day morality (Figure 3) showed that mortality rates increased with higher BMI categories (log-rank *P <* 0.001). The severe obesity group had the highest 30-day mortality (54.8%), whereas the underweight group had the lowest (24.3%). Multivariate Cox regression analysis, treating BMI as a continuous variable, indicated an independent association between BMI and mortality (aHR: 1.06 [95% CI: 1.05-1.08] per 1.0 kg/m^2^ increase in BMI; *P <* 0.001). In the Cox regression analysis treating BMI as a category, aHR (using normal weight as reference) was 0.71 (95% CI: 0.56-0.90; *P =* 0.005) for underweight, 1.03 (95% CI: 0.88-1.21; *P =* 0.681) for overweight, 1.37 (95% CI: 1.19-1.57; *P <* 0.001) for obesity, and 2.00 (95% CI: 1.66-2.41; *P <* 0.001) for severe obesity (Central Illustration). A sensitivity analysis including other variables with a significant difference among BMI categories as covariates confirmed the same effect of BMI (Supplemental Table 2).

**Table 2:** 30-Day Mortality and Adverse Events According to Body Mass Index Categories

	Overall (N = 3,636)	Underweight (n = 338)	Normal Weight (n = 1,383)	Overweight (n = 705)	Obesity (n = 927)	Severe Obesity (n = 283)	*P* Value
30-d mortality	1,306/3,636 (35.9)	81/338 (24.0)	441/1,383 (31.9)	245/705 (34.8)	385/927 (41.5)	154/283 (54.4)	<0.001
Adverse events							
Bleeding	832/3,636 (22.9)	90/338 (26.6)	312/1,383 (22.6)	159/705 (22.6)	197/927 (21.3)	74/283 (26.1)	0.205
Hemolysis	532/3,636 (14.6)	37/338 (10.9)	184/1,383 (13.3)	122/705 (17.3)	140/927 (15.1)	49/283 (17.3)	0.022
Lower limb ischemia	171/3,636 (4.7)	11/338 (3.3)	88/1,383 (6.4)	27/705 (3.8)	33/927 (3.6)	12/283 (4.2)	0.007
Access-related vascular injury	52/3,636 (1.4)	6/338 (1.8)	18/1,383 (1.3)	11/705 (1.6)	13/927 (1.4)	4/283 (1.4)	0.941
Cerebrovascular accident							
Ischemic stroke	134/3,636 (3.7)	11/338 (3.3)	43/1,383 (3.1)	29/705 (4.1)	40/927 (4.3)	11/283 (3.9)	0.574
Hemorrhagic stroke	85/3,636 (2.3)	1/338 (0.3)	27/1,383 (2.0)	20/705 (2.8)	26/927 (2.8)	11/283 (3.9)	0.020

Values are n/N (%).

**Central Illustration:**
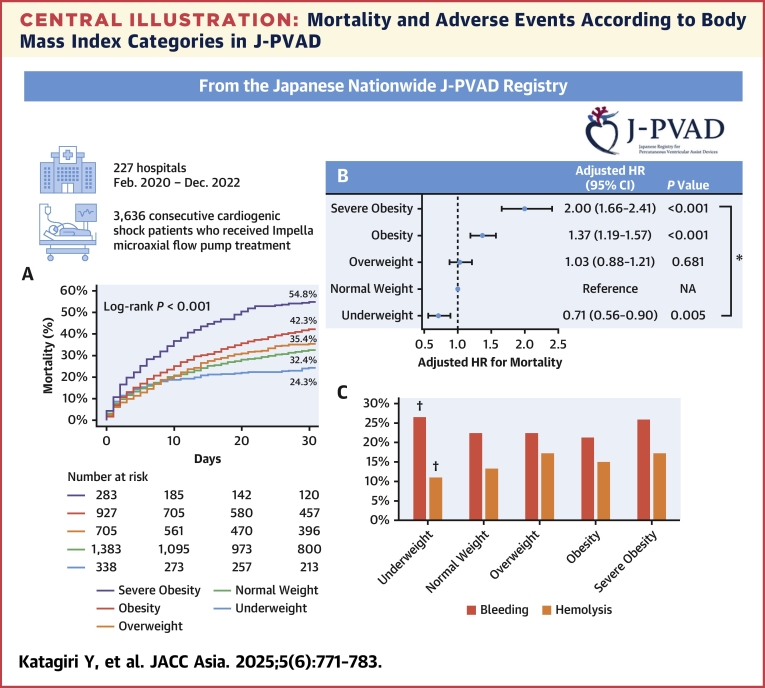
Mortality and Adverse Events According to Body Mass Index Categories in J-PVAD From the J-PVAD (Japanese Registry for Percutaneous Ventricular Assist Device), patients with cardiogenic shock treated with microaxial flow pumps were stratified into 5 body mass index categories: underweight (<18.5 kg/m^2^), normal weight (18.5-22.9 kg/m^2^), overweight (23.0-24.9 kg/m^2^), obesity (25.0-29.9 kg/m^2^), and severe obesity (≥30.0 kg/m^2^).^15^ (A) Kaplan-Meier estimates and (B) adjusted HR for mortality. Severe obesity and obesity were associated with a higher risk of mortality, whereas underweight was associated with a lower risk of mortality, compared with normal weight. ∗Overall *P <* 0.001. (C) The incidence of bleeding and hemolysis. Bleeding was numerically more frequent in underweight and severely obese groups *(P =* 0.205), whereas hemolysis increased with higher body mass index *(P =* 0.022). † indicates association with subsequent mortality. NA = not applicable.

**Figure 4:**
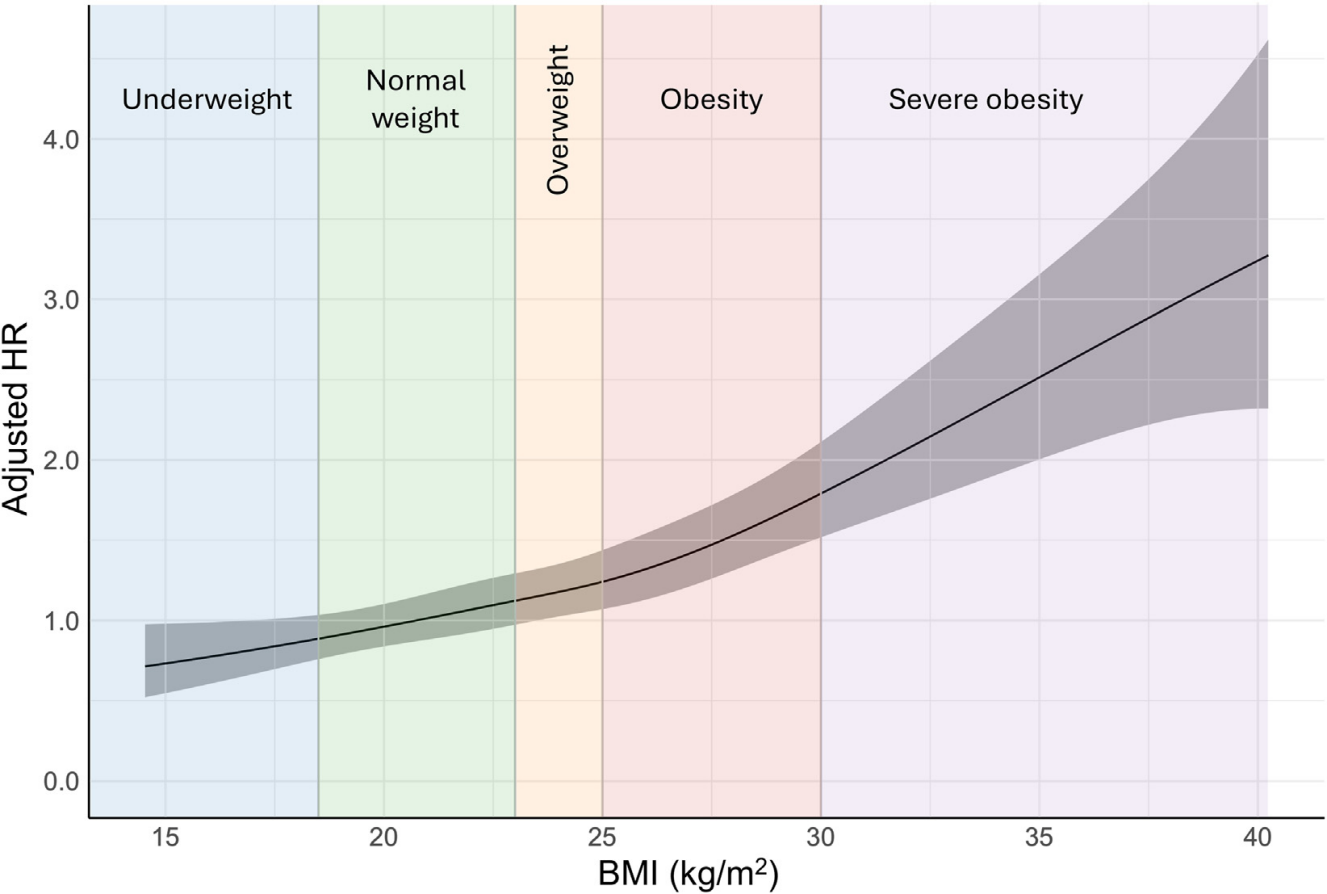
Restricted Cubic Spline Curve Analysis of BMI Predicting Mortality The restricted cubic spline curve plotting adjusted HR of mortality on the y-axis and body mass index (BMI) on the x-axis, constructed with knots at the borderline of the BMI categories. The center of the normal weight range was set as a reference. The gray area represents the 95% CI.

**Figure 5:**
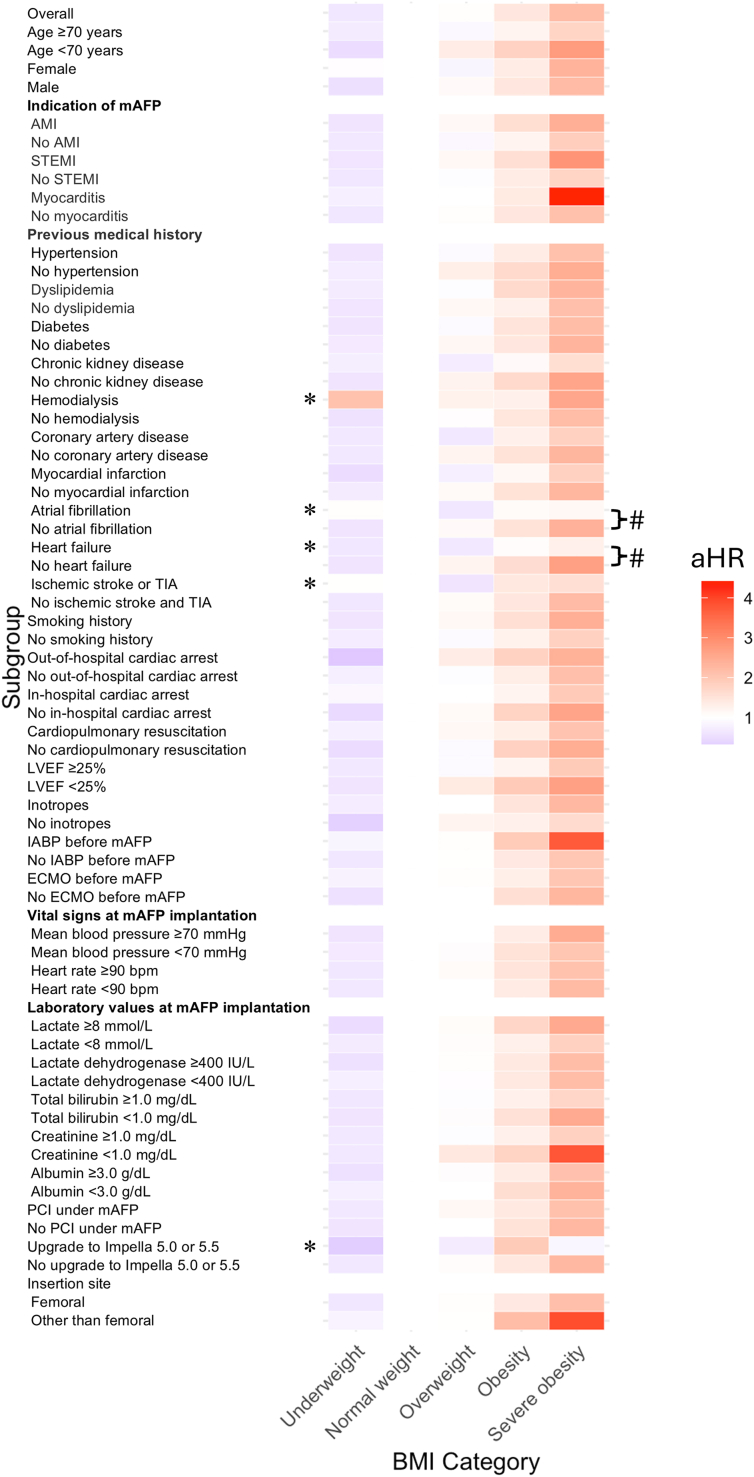
Heatmap of aHR According to BMI Categories in Various Subgroups The heatmap illustrates adjusted hazard ratio (aHR) for 30-day mortality across body mass index (BMI) categories within different subgroups. In the color code, white represents the reference of aHR = 1.0 in normal weight, blue indicates lower aHR, and red indicates higher aHR than reference. ∗*P* for trend ≥0.05. All other subgroups without ∗ indicate *P* for trend <0.05. #*P* for interaction <0.05 (marked only if either subgroup has *P* for trend ≥0.05). AMI = acute myocardial infarction; ECMO = extracorporeal membrane oxygenation; IABP = intra-aortic balloon pump; LVEF = left ventricular ejection fraction; mAFP = microaxial flow pump; PCI = percutaneous coronary intervention; STEMI = ST-segment elevation myocardial infarction; TIA = transient ischemic attack.

Spline curve analysis indicated a positive linear relationship between BMI and mortality without a U-shaped pattern (Figure 4). A comprehensive subgroup analysis demonstrated consistent results across various patient subgroups (Figure 5, Supplemental Table 3), with increased aHR observed in higher BMI categories, except in patients with chronic hemodialysis, atrial fibrillation, a history of heart failure, ischemic stroke including transient ischemic attack, or upgrade to Impella 5.0 or 5.5 (*P* for trend ≥0.05). Notably, among these subgroups, the interaction between BMI categories and comorbidities was significant only in patients with atrial fibrillation or a history of heart failure, indicating a marked absence of increase in aHR with higher BMI categories observed in those without these conditions.

### BMI and adverse events

The incidence of adverse events across BMI categories is summarized in Table 2. Overall, bleeding and hemolysis were the most frequent adverse events. Bleeding rates did not differ significantly among BMI categories, although they were numerically more frequent in patients who were underweight and severely obese *(P =* 0.205). Table 3 presents the incidence of bleeding in subgroups of patients likely to be on dual antiplatelet therapy. In patients who underwent percutaneous coronary intervention (PCI) under mAFP, the incidence of bleeding was significantly different among BMI categories, following a U-shaped pattern: highest in underweight and severely obese groups and lowest in patients who were overweight. Hemolysis was more frequent in overweight and severely obese groups (both 17.3%) and less frequent in patients who were underweight (10.9%) (Table 2).

**Table 3:** Comparison of Bleeding Event Incidence Among Patients With a High Expected Prevalence of DAPT

	Overall	Underweight	Normal weight	Overweight	Obesity	Severe obesity	*P* Value
Etiology of CS: AMI	529/2,263 (23.4)	50/182 (27.5)	194/822 (23.6)	105/484 (21.7)	131/611 (21.4)	49/164 (29.9)	0.106
Etiology of CS: STEMI	428/1,808 (23.7)	42/148 (28.4)	156/652 (23.9)	83/390 (21.3)	106/491 (21.6)	41/127 (32.3)	0.051
History of MI	141/685 (20.6)	12/53 (22.6)	48/248 (19.4)	27/141 (19.1)	38/184 (20.7)	16/59 (27.1)	0.720
PCI under mAFP	380/1,641 (23.2)	43/140 (30.7)	146/611 (23.9)	71/363 (19.6)	88/425 (20.7)	32/102 (31.4)	0.013

Values are n/N (%).

CS = cardiogenic shock; DAPT = dual antiplatelet therapy; STEMI = ST-segment elevation myocardial infarction; other abbreviations as in Table 1.

Interestingly, lower limb ischemia was least frequent in underweight patients. The rates of access-related vascular injury and ischemic stroke showed no significant differences among BMI categories. Hemorrhagic stroke occurred most frequently in patients who were severely obese (3.9%), whereas only 1 (0.3%) patient in the underweight group experienced it.

### Impact of bleeding and hemolysis on mortality

Table 4 shows the effect of bleeding and hemolysis on mortality using a time-updated Cox regression model. In the overall population, bleeding was independently associated with mortality, whereas hemolysis was not. When stratified by BMI categories, both bleeding and hemolysis were independently associated with mortality only in the underweight group (aHR: 3.20 [95% CI: 1.95-5.25]; *P <* 0.001 for bleeding, and aHR: 2.19 [95% CI: 1.06-4.51]; *P =* 0.034 for hemolysis).

**Table 4:** Impact of Bleeding and Hemolysis on Mortality

	Overall			Underweight			Normal Weight			Overweight			Obesity			Severe Obesity		
	aHR	95% CI	*P* Value	aHR	95% CI	*P* Value	aHR	95% CI	*P* Value	aHR	95% CI	*P* Value	aHR	95% CI	*P* Value	aHR	95% CI	*P* Value
Bleeding	1.20	1.05-1.37	0.006	3.20	1.95-5.25	<0.001	1.03	0.82-1.30	0.805	1.28	0.94-1.73	0.117	1.18	0.91-1.51	0.210	1.12	0.76-1.63	0.570
Hemolysis	1.09	0.93-1.29	0.281	2.19	1.06-4.51	0.034	0.99	0.74-1.33	0.948	1.16	0.83-1.64	0.389	1.10	0.82-1.49	0.514	0.86	0.51-1.44	0.558

Bleeding and hemolysis were included as time-updated covariates in the multivariate Cox regression model, which was adjusted for body mass index, age, sex, etiology of cardiogenic shock, in-hospital cardiac arrest, extracorporeal membrane oxygenation use, mean blood pressure, lactate, lactate dehydrogenase, total bilirubin, creatinine, and albumin.

aHR = adjusted HR.

## Discussion

In this study evaluating the clinical impact of BMI in patients with CS treated with mAFP in the J-PVAD registry, the major findings were as follows:

1. Obesity and severe obesity were independently associated with increased 30-day mortality, whereas underweight was associated with better survival compared with normal weight.
2. A spline curve analysis indicated no U-shaped relationship between BMI and adjusted risk of 30-day mortality; instead, a positive linear association was observed.
3. Most subgroups consistently showed an association between increased BMI and increased mortality.
4. Although the rate of bleeding did not differ among BMI categories in the overall population, underweight and severe obesity were associated with bleeding complications in patients who underwent PCI under mAFP. Hemolysis increased in higher BMI categories.
5. Bleeding and hemolysis were independently associated with mortality only in patients who were underweight.

Given the limited data on the impact of BMI in patients treated with MCS, the J-PVAD registry provided a unique opportunity to explore this topic. To the best of our knowledge, this is the first report to comprehensively investigate the association between BMI and clinical outcomes in patients with CS treated with mAFP. The linear relationship between BMI and mortality contrasts with the "obesity paradox" observed in some heart failure^7^^,^^8^ and ischemic heart disease studies,^9^^,^^10^ aligning with the previous study in patients with CS treated with acute mechanical supports.^11^ A higher mortality in more obese patients may be attributed to baseline critical conditions, as indicated by the higher frequency of cardiac arrest and ECMO use before mAFP implantation—both of which have been reported to be associated with increased mortality.^16^ However, given the independence of the association between higher BMI and mortality, higher body mass itself would contribute to the increased risk of mortality. In the present study, most patients (∼93%) began treatment with the Impella CP, regardless of BMI category. Although Impella CP is commonly used for first-line mAFP, it may not provide adequate cardiac unloading and end-organ perfusion in some patients with higher BMI. In such cases, MCS upgrade should be considered. Supplemental Table 4 demonstrates significantly better survival in severely obese patients who underwent an Impella upgrade to 5.0 or 5.5 compared with those who did not upgrade from 2.5 or CP; however, this finding requires cautious interpretation because of the limited number of observations and the retrospective nature of the analysis. Nevertheless, the frequency of mAFP upgrade was only 3.9% in the present population. Contrarily, better survival in patients with lower BMI may reflect a relatively higher support flow provided by Impella CP.

The comprehensive subgroup analysis, including different CS etiologies, showed an overall consistent relationship between BMI and mortality risk. However, interestingly, in patients with atrial fibrillation or heart failure, the trend of increased mortality in higher BMI categories was absent. Generally, both atrial fibrillation^18^ and heart failure^19^ result in a decrease in cardiac output. Chronic exposure to such conditions before the CS event, possibly combined with cardioprotective medications, may provide preconditioning to CS.

Bleeding and hemolysis complications are important concerns in the management of mAFP.^12^ Although previous studies have shown a higher incidence of bleeding in patients who are underweight after ECMO^20^ or those with obesity after MCS,^11^ the incidence of bleeding did not differ among BMI categories in the overall population in the present study. This may reflect the precise anticoagulation management by titration of heparin through monitoring activated clotting time, as routinely recommended by the manufacturer. However, in patients who underwent PCI under mAFP, a subgroup at high risk of bleeding caused by dual antiplatelet therapy and anticoagulation, both underweight and severe obesity were associated with bleeding events. Previous studies^21^^,^^22^ and the fact that the J-HBR criteria,^23^ which were developed to predict bleeding risk in patients after PCI, include low body weight besides the original ARC-HBR criteria,^24^ support the bleeding vulnerability in underweight patients with intense antiplatelet therapy. On the other hand, the technical difficulty in percutaneous insertion of mAFP in patients who are severely obese could result in subsequent bleeding complications, which could be exaggerated by intense antiplatelet therapy. Complete single antiplatelet therapy after PCI is appealing and could be a reasonable choice for these patients, especially during the acute phase up to 30 days after mAFP insertion, pending confirmation of its safety and efficacy in ongoing trials.^25^^,^^26^ Increased hemolysis in patients with higher BMI categories could be attributed to the higher support levels required to maintain adequate cardiac output compared with those who are underweight. Although previous studies did not indicate the mortality impact of bleeding^27^ and hemolysis^28^ in patients treated with mAFP, the present analysis revealed that bleeding and hemolysis were associated with increased mortality in patients who were underweight. This may be related to underlying frailty and limited physiological reserves to withstand complications in underweight patients. Although the incidence of bleeding and hemolysis was not high in underweight patients, appropriate measures, such as hemostasis and anticoagulation management for bleeding and pump positioning and support level adjustment for hemolysis, are important in these patients.^12^ Lower limb ischemia was least frequent in underweight patients, likely because they had fewer atherosclerotic risk factors, preventing significant peripheral atherosclerosis progression. The acquired von Willebrand syndrome could explain the increased incidence of hemorrhagic stroke in the higher BMI category, as these patients require higher support flow, although this did not translate into an increase in the overall bleeding rate.

Our findings highlight the importance of considering BMI as a risk-stratifying factor in patients with CS requiring mAFP support. BMI-specific management strategies should be implemented through a multidisciplinary evaluation before and after the initiation of mAFP treatment. Patients with obesity, caused by their higher mortality risk, may benefit from close monitoring and early upgrade of hemodynamic support to reduce hemolysis. On the other hand, underweight patients may require proactive measures to prevent bleeding and hemolysis, as these adverse events are independently associated with increased mortality.

### Study limitations

First, the registry only included patients with CS treated with mAFP, and the lack of a comparator device made comparisons with other devices difficult. Second, selection bias can exist, because the decision of the attending physician to initiate mAFP treatment could have been influenced by underweight or obesity, considering basal nutrition status or complications during intensive care. Third, no standardized protocols for managing patients with CS were used across centers, which may have introduced variability in patient management and outcomes. Fourth, BMI was assessed only at admission. Overestimation of BMI might have occurred in cases of volume overload in congestive heart failure. Additionally, changes in body weight after admission were not considered. Fifth, although ischemic heart disease, including AMI and ischemic cardiomyopathy, was the dominant cause of CS in the present study, the details of coronary artery disease were not captured. Sixth, the absence of information on medical therapy precluded analyses or statistical adjustments for antiplatelet, anticoagulation, and optimal medical therapy after heart failure. Finally, this study was based on retrospective analyses, and the findings should be interpreted as hypothesis-generating. Prospective studies are warranted to investigate BMI-specific management in patients with CS undergoing mAFP therapy. More specifically, future studies should focus on the utility of BMI-specific intensified monitoring of complications, and the effectiveness of early escalation of mAFP to prevent hemolysis in underweight patients or to improve survival in obese patients.

## Conclusions

Higher BMI was associated with elevated mortality in patients with CS requiring mAFP support without exhibiting a U-shaped mortality relationship. The incidence of bleeding was higher in underweight and severely obese groups after PCI under mAFP, whereas hemolysis was more frequent in higher BMI categories. Bleeding and hemolysis were associated with mortality only in underweight patients. These findings highlight the need for BMI-specific management strategies in patients with CS undergoing mAFP therapy.

## Funding Support and Author Disclosures

Drs Katagiri and Ikeda have received honoraria from Abiomed Japan. Dr Sotomi has received grants from Abiomed. All other authors have reported that they have no relationships relevant to the contents of this paper to disclose.
