# Supplementary material for: Effect of Body Mass Index in Patients With Cardiogenic Shock Requiring Microaxial Flow Pump
Source: JACC Asia. 2025 Mar 24;5(6):771–83. doi: 10.1016/j.jacasi.2025.03.003 (PMC12287744; doi:10.1016/j.jacasi.2025.03.003)
Supplement: Supplemental Tables 1-4 [file mmc1.docx]

**Supplemental Material**

**Supplemental Table 1. Missing numbers of baseline variables.**

|  | **Overall** | **Underweight** | **Normal weight** | **Overweight** | **Obesity** | **Severe obesity** |
| --- | --- | --- | --- | --- | --- | --- |
|  | n = 3,636 | n = 338 | n = 1,383 | n = 705 | n = 927 | n = 283 |
| Smoking | 545 (15.0%) | 45 (13.3%) | 184 (13.3%) | 102 (14.5%) | 160 (17.3%) | 54 (19.1%) |
| Out-of-hospital cardiac arrest | 25 (0.7%) | 2 (0.6%) | 10 (0.7%) | 5 (0.7%) | 5 (0.5%) | 3 (1.1%) |
| Left ventricular ejection fraction | 1,718 (47.2%) | 134 (39.6%) | 645 (46.6%) | 331 (47.0%) | 473 (51.0%) | 135 (47.7%) |
| **Previous medical history** |  |  |  |  |  |  |
| Hypertension | 194 (5.3%) | 13 (3.8%) | 81 (5.9%) | 30 (4.3%) | 53 (5.7%) | 17 (6.0%) |
| Dyslipidemia | 188 (5.2%) | 11 (3.3%) | 75 (5.4%) | 30 (4.3%) | 55 (5.9%) | 17 (6.0%) |
| Diabetes | 152 (4.2%) | 11 (3.3%) | 65 (4.7%) | 24 (3.4%) | 38 (4.1%) | 14 (4.9%) |
| Chronic kidney disease | 184 (5.1%) | 10 (3.0%) | 71 (5.1%) | 34 (4.8%) | 51 (5.5%) | 18 (6.4%) |
| Hemodialysis | 186 (5.1%) | 10 (3.0%) | 72 (5.2%) | 34 (4.8%) | 52 (5.6%) | 18 (6.4%) |
| Coronary artery disease | 177 (4.9%) | 16 (4.7%) | 64 (4.6%) | 29 (4.1%) | 48 (5.2%) | 20 (7.1%) |
| Myocardial infarction | 162 (4.5%) | 10 (3.0%) | 61 (4.4%) | 28 (4.0%) | 45 (4.9%) | 18 (6.4%) |
| Heart failure | 170 (4.7%) | 13 (3.8%) | 64 (4.6%) | 24 (3.4%) | 51 (5.5%) | 18 (6.4%) |
| Ischemic stroke or TIA | 144 (4.0%) | 8 (2.4%) | 50 (3.6%) | 25 (3.5%) | 44 (4.7%) | 17 (6.0%) |
| **Vital signs at mAFP implantation** |  |  |  |  |  |  |
| Diastolic blood pressure | 1 (0.0%) | 0 (0.0%) | 1 (0.1%) | 0 (0.0%) | 0 (0.0%) | 0 (0.0%) |
| Mean blood pressure | 1 (0.0%) | 0 (0.0%) | 1 (0.1%) | 0 (0.0%) | 0 (0.0%) | 0 (0.0%) |
| **Laboratory values at mAFP implantation** |  |  |  |  |  |  |
| Lactate | 811 (22.3%) | 90 (26.6%) | 303 (21.9%) | 172 (24.4%) | 196 (21.1%) | 50 (17.7%) |
| Lactate dehydrogenase | 237 (6.5%) | 23 (6.8%) | 105 (7.6%) | 38 (5.4%) | 53 (5.7%) | 18 (6.4%) |
| Total bilirubin | 229 (6.3%) | 22 (6.5%) | 92 (6.7%) | 38 (5.4%) | 63 (6.8%) | 14 (4.9%) |
| Albumin | 236 (6.5%) | 23 (6.8%) | 96 (6.9%) | 40 (5.7%) | 62 (6.7%) | 15 (5.3%) |
| Creatinine | 61 (1.7%) | 7 (2.1%) | 24 (1.7%) | 11 (1.6%) | 16 (1.7%) | 3 (1.1%) |

Data are presented as numbers (percentage).

Among the variables shown in **Table 1**, those not included in this table have no missing values.

mAFP = microaxial flow pump; TIA = transient ischemic attack.

**Supplemental Table 2. Sensitivity analysis of the multivariate Cox regression models predicting 30-day mortality.**

|  | **Model 1** | | | **Model 2** | | | **Model 3** | | | **Model 4** | | |
| --- | --- | --- | --- | --- | --- | --- | --- | --- | --- | --- | --- | --- |
|  | HR | 95% CI | P value | HR | 95% CI | P value | HR | 95% CI | P value | HR | 95% CI | P value |
| BMI category |  |  |  | - | | |  |  |  | - | | |
| Underweight | 0.71 | 0.56–0.90 | 0.005 |  |  |  | 0.71 | 0.56–0.90 | 0.004 |  |  |  |
| Normal weight | Reference | | NA |  |  |  | Reference | | NA |  |  |  |
| Overweight | 1.03 | 0.88–1.21 | 0.681 |  |  |  | 1.04 | 0.89–1.22 | 0.624 |  |  |  |
| Obesity | 1.37 | 1.19–1.57 | <0.001 |  |  |  | 1.39 | 1.21–1.60 | <0.001 |  |  |  |
| Severe obesity | 2.00 | 1.66–2.41 | <0.001 |  |  |  | 1.97 | 1.63–2.39 | <0.001 |  |  |  |
| BMI (per 1 kg/m^2^ increase) | - | | | 1.06 | 1.05–1.08 | <0.001 | - | | | 1.06 | 1.05–1.08 | <0.001 |

In addition to BMI (either as a categorical [**Models 1** and **3**] or continuous variable [**Models 2** and **4**]), the following adjusting variables were included as covariates in each model predicting 30-day mortality. **Models 1** and **2**: age, sex, etiology of CS, in-hospital cardiac arrest, use of ECMO, mean blood pressure, lactate, lactate dehydrogenase, total bilirubin, creatinine, and albumin. **Models 3** and **4**: in addition to adjusting covariates in Models 1 and 2, hypertension, dyslipidemia, diabetes, heart failure, smoking, out-of-hospital cardiac arrest, cardiopulmonary resuscitation, PCI under mAFP, mAFP device, and insertion site.

BMI = body mass index; CI = confidence interval; CS = cardiogenic shock; ECMO = extracorporeal membrane oxygenation; HR = hazard ratio; mAFP = microaxial flow pump; NA = not applicable; PCI = percutaneous coronary intervention.

**Supplemental Table 3. Adjusted hazard ratios of 30-day mortality in the various subgroups.**

|  | **Underweight** | | | **Normal weight** | | | **Overweight** | | | **Obesity** | | | **Severe obesity** | | | P for trend | P for interaction |
| --- | --- | --- | --- | --- | --- | --- | --- | --- | --- | --- | --- | --- | --- | --- | --- | --- | --- |
|  | aHR | 95% CI | P value | aHR | 95% CI | P value | aHR | 95% CI | P value | aHR | 95% CI | P value | aHR | 95% CI | P value |  |  |
| Overall | 0.71 | 0.56–0.90 | 0.005 | Reference | | NA | 1.03 | 0.88–1.21 | 0.681 | 1.37 | 1.19–1.57 | <0.001 | 2.00 | 1.66–2.41 | <0.001 | <0.001 | NA |
| Age ≥70 years | 0.74 | 0.56–0.98 | 0.034 | Reference | | NA | 0.90 | 0.73–1.10 | 0.298 | 1.18 | 0.97–1.43 | 0.102 | 1.64 | 1.21–2.24 | 0.002 | <0.001 | 0.005 |
| Age <70 years | 0.59 | 0.36–0.96 | 0.033 | Reference | | NA | 1.29 | 1.00–1.66 | 0.050 | 1.67 | 1.36–2.06 | <0.001 | 2.47 | 1.92–3.19 | <0.001 | <0.001 |  |
| Female | 0.97 | 0.66–1.44 | 0.885 | Reference | | NA | 0.87 | 0.60–1.26 | 0.459 | 1.29 | 0.95–1.76 | 0.099 | 2.13 | 1.41–3.21 | <0.001 | 0.002 | 0.166 |
| Male | 0.61 | 0.45–0.83 | 0.001 | Reference | | NA | 1.08 | 0.91–1.29 | 0.370 | 1.36 | 1.16–1.60 | <0.001 | 2.01 | 1.62–2.49 | <0.001 | <0.001 |  |
| **Indication of mAFP** |  |  |  |  |  |  |  |  |  |  |  |  |  |  |  |  |  |
| AMI | 0.67 | 0.48–0.92 | 0.013 | Reference | | NA | 1.10 | 0.91–1.33 | 0.329 | 1.50 | 1.27–1.77 | <0.001 | 2.19 | 1.73–2.77 | <0.001 | <0.001 | 0.429 |
| No AMI | 0.73 | 0.51–1.04 | 0.083 | Reference | | NA | 0.89 | 0.66–1.20 | 0.447 | 1.17 | 0.91–1.50 | 0.226 | 1.73 | 1.27–2.37 | <0.001 | <0.001 |  |
| STEMI | 0.69 | 0.48–0.99 | 0.042 | Reference | | NA | 1.11 | 0.90–1.38 | 0.335 | 1.49 | 1.22–1.82 | <0.001 | 2.57 | 1.95–3.37 | <0.001 | <0.001 | 0.485 |
| No STEMI | 0.69 | 0.50–0.96 | 0.025 | Reference | | NA | 0.95 | 0.75–1.19 | 0.647 | 1.30 | 1.07–1.58 | 0.009 | 1.64 | 1.26–2.13 | <0.001 | <0.001 |  |
| Myocarditis | 0.80 | 0.32–1.97 | 0.625 | Reference | | NA | 0.99 | 0.47–2.07 | 0.983 | 1.32 | 0.72–2.43 | 0.371 | 3.87 | 1.72–8.69 | 0.001 | 0.009 | 0.845 |
| No myocarditis | 0.71 | 0.55–0.91 | 0.006 | Reference | | NA | 1.04 | 0.88–1.22 | 0.656 | 1.36 | 1.18–1.57 | <0.001 | 1.94 | 1.60–2.36 | <0.001 | <0.001 |  |
| **Previous medical history** |  |  |  |  |  |  |  |  |  |  |  |  |  |  |  |  |  |
| Hypertension | 0.67 | 0.49–0.91 | 0.009 | Reference | | NA | 0.92 | 0.75–1.13 | 0.422 | 1.29 | 1.09–1.54 | 0.004 | 1.94 | 1.54–2.45 | <0.001 | <0.001 | 0.462 |
| No hypertension | 0.75 | 0.51–1.11 | 0.148 | Reference | | NA | 1.25 | 0.97–1.62 | 0.083 | 1.55 | 1.23–1.96 | <0.001 | 2.23 | 1.58–3.13 | <0.001 | <0.001 |  |
| Dyslipidemia | 0.74 | 0.51–1.08 | 0.120 | Reference | | NA | 0.96 | 0.75–1.23 | 0.730 | 1.55 | 1.26–1.91 | <0.001 | 2.11 | 1.59–2.81 | <0.001 | <0.001 | 0.231 |
| No dyslipidemia | 0.69 | 0.50–0.94 | 0.017 | Reference | | NA | 1.11 | 0.90–1.36 | 0.329 | 1.25 | 1.03–1.51 | 0.024 | 1.96 | 1.51–2.53 | <0.001 | <0.001 |  |
| Diabetes | 0.67 | 0.46–0.99 | 0.044 | Reference | | NA | 0.92 | 0.71–1.19 | 0.525 | 1.41 | 1.13–1.75 | 0.002 | 1.99 | 1.50–2.63 | <0.001 | <0.001 | 0.658 |
| No diabetes | 0.74 | 0.54–1.00 | 0.049 | Reference | | NA | 1.13 | 0.92–1.38 | 0.237 | 1.36 | 1.13–1.64 | 0.001 | 2.11 | 1.62–2.75 | <0.001 | <0.001 |  |
| Chronic kidney disease | 0.78 | 0.55–1.13 | 0.189 | Reference | | NA | 0.76 | 0.58–1.00 | 0.048 | 1.09 | 0.86–1.39 | 0.479 | 1.50 | 1.08–2.08 | 0.015 | 0.015 | 0.006 |
| No chronic kidney disease | 0.66 | 0.48–0.91 | 0.011 | Reference | | NA | 1.20 | 0.99–1.46 | 0.065 | 1.56 | 1.32–1.86 | <0.001 | 2.34 | 1.85–2.96 | <0.001 | <0.001 |  |
| Hemodialysis | 1.92 | 0.94–3.92 | 0.073 | Reference | | NA | 1.20 | 0.59–2.46 | 0.612 | 1.23 | 0.65–2.33 | 0.532 | 2.32 | 1.00–5.35 | 0.049 | 0.594 | 0.051 |
| No hemodialysis | 0.64 | 0.50–0.83 | <0.001 | Reference | | NA | 1.03 | 0.87–1.21 | 0.748 | 1.38 | 1.20–1.59 | <0.001 | 2.00 | 1.65–2.43 | <0.001 | <0.001 |  |
| Coronary artery disease | 0.72 | 0.47–1.10 | 0.127 | Reference | | NA | 0.73 | 0.54–1.00 | 0.048 | 1.22 | 0.94–1.59 | 0.142 | 1.71 | 1.21–2.41 | 0.002 | <0.001 | 0.093 |
| No coronary artery disease | 0.73 | 0.55–0.97 | 0.032 | Reference | | NA | 1.17 | 0.97–1.41 | 0.094 | 1.43 | 1.21–1.68 | <0.001 | 2.09 | 1.66–2.62 | <0.001 | <0.001 |  |
| Myocardial infarction | 0.58 | 0.33–1.03 | 0.062 | Reference | | NA | 0.79 | 0.55–1.13 | 0.204 | 1.11 | 0.80–1.52 | 0.535 | 1.70 | 1.13–2.55 | 0.010 | 0.003 | 0.420 |
| No myocardial infarction | 0.74 | 0.57–0.97 | 0.026 | Reference | | NA | 1.09 | 0.92–1.31 | 0.314 | 1.44 | 1.23–1.68 | <0.001 | 2.08 | 1.67–2.58 | <0.001 | <0.001 |  |
| Atrial fibrillation | 1.03 | 0.58–1.85 | 0.909 | Reference | | NA | 0.70 | 0.40–1.22 | 0.211 | 1.08 | 0.68–1.71 | 0.739 | 1.12 | 0.53–2.34 | 0.772 | 0.915 | 0.014 |
| No atrial fibrillation | 0.66 | 0.51–0.86 | 0.002 | Reference | | NA | 1.08 | 0.92–1.28 | 0.333 | 1.42 | 1.23–1.64 | <0.001 | 2.16 | 1.77–2.63 | <0.001 | <0.001 |  |
| Heart failure | 0.71 | 0.48–1.05 | 0.084 | Reference | | NA | 0.72 | 0.52–1.00 | 0.050 | 1.06 | 0.79–1.42 | 0.704 | 1.24 | 0.86–1.79 | 0.253 | 0.071 | 0.008 |
| No heart failure | 0.68 | 0.50–0.92 | 0.012 | Reference | | NA | 1.16 | 0.97–1.39 | 0.112 | 1.52 | 1.30–1.79 | <0.001 | 2.41 | 1.93–3.01 | <0.001 | <0.001 |  |
| Ischemic stroke or TIA | 1.02 | 0.48–2.16 | 0.961 | Reference | | NA | 0.66 | 0.36–1.20 | 0.177 | 1.35 | 0.85–2.14 | 0.203 | 1.48 | 0.70–3.12 | 0.304 | 0.177 | 0.369 |
| No ischemic stroke and TIA | 0.70 | 0.54–0.90 | 0.005 | Reference | | NA | 1.08 | 0.92–1.27 | 0.362 | 1.37 | 1.18–1.58 | <0.001 | 2.01 | 1.66–2.45 | <0.001 | <0.001 |  |
| Smoking history | 0.66 | 0.47–0.93 | 0.016 | Reference | | NA | 1.10 | 0.91–1.34 | 0.319 | 1.48 | 1.24–1.77 | <0.001 | 2.18 | 1.74–2.75 | <0.001 | <0.001 | 0.295 |
| No smoking history | 0.76 | 0.54–1.07 | 0.111 | Reference | | NA | 0.93 | 0.71–1.21 | 0.581 | 1.21 | 0.95–1.53 | 0.115 | 1.69 | 1.20–2.38 | 0.003 | <0.001 |  |
| Out-of-hospital cardiac arrest | 0.33 | 0.16–0.70 | 0.004 | Reference | | NA | 1.28 | 0.96–1.71 | 0.097 | 1.67 | 1.30–2.16 | <0.001 | 2.16 | 1.56–3.00 | <0.001 | <0.001 | 0.020 |
| No out-of-hospital cardiac arrest | 0.79 | 0.61–1.02 | 0.069 | Reference | | NA | 0.95 | 0.79–1.15 | 0.618 | 1.27 | 1.07–1.50 | 0.005 | 1.95 | 1.54–2.47 | <0.001 | <0.001 |  |
| In-hospital cardiac arrest | 0.89 | 0.63–1.26 | 0.514 | Reference | | NA | 1.01 | 0.80–1.27 | 0.944 | 1.17 | 0.95–1.44 | 0.140 | 1.79 | 1.37–2.34 | <0.001 | <0.001 | 0.024 |
| No in-hospital cardiac arrest | 0.54 | 0.39–0.76 | <0.001 | Reference | | NA | 1.10 | 0.89–1.36 | 0.398 | 1.66 | 1.37–2.00 | <0.001 | 2.34 | 1.79–3.07 | <0.001 | <0.001 |  |
| Cardiopulmonary resuscitation | 0.81 | 0.59–1.11 | 0.190 | Reference | | NA | 1.12 | 0.92–1.37 | 0.271 | 1.26 | 1.05–1.51 | 0.012 | 1.90 | 1.51–2.39 | <0.001 | <0.001 | 0.141 |
| No cardiopulmonary resuscitation | 0.58 | 0.40–0.84 | 0.004 | Reference | | NA | 0.91 | 0.70–1.18 | 0.480 | 1.68 | 1.35–2.10 | <0.001 | 2.23 | 1.58–3.13 | <0.001 | <0.001 |  |
| LVEF ≥25% | 0.72 | 0.55–0.96 | 0.022 | Reference | | NA | 0.91 | 0.74–1.12 | 0.395 | 1.18 | 0.98–1.43 | 0.083 | 1.78 | 1.36–2.34 | <0.001 | <0.001 | 0.016 |
| LVEF <25% | 0.66 | 0.41–1.06 | 0.084 | Reference | | NA | 1.32 | 1.03–1.70 | 0.027 | 1.80 | 1.45–2.23 | <0.001 | 2.40 | 1.83–3.15 | <0.001 | <0.001 |  |
| Inotropes | 0.76 | 0.59–0.98 | 0.037 | Reference | | NA | 1.01 | 0.85–1.20 | 0.935 | 1.41 | 1.21–1.64 | <0.001 | 2.05 | 1.67–2.51 | <0.001 | <0.001 | 0.202 |
| No inotropes | 0.43 | 0.22–0.87 | 0.018 | Reference | | NA | 1.16 | 0.81–1.67 | 0.424 | 1.24 | 0.88–1.74 | 0.224 | 1.54 | 0.91–2.60 | 0.110 | 0.003 |  |
| IABP before mAFP | 0.86 | 0.40–1.88 | 0.709 | Reference | | NA | 1.04 | 0.60–1.80 | 0.897 | 1.77 | 1.13–2.76 | 0.013 | 3.30 | 1.92–5.64 | <0.001 | <0.001 | 0.289 |
| No IABP before mAFP | 0.70 | 0.54–0.90 | 0.005 | Reference | | NA | 1.02 | 0.86–1.20 | 0.813 | 1.34 | 1.16–1.55 | <0.001 | 1.85 | 1.51–2.26 | <0.001 | <0.001 |  |
| ECMO before mAFP | 0.83 | 0.56–1.23 | 0.345 | Reference | | NA | 1.04 | 0.81–1.32 | 0.776 | 1.26 | 1.02–1.55 | 0.035 | 1.85 | 1.43–2.40 | <0.001 | <0.001 | 0.647 |
| No ECMO before mAFP | 0.64 | 0.47–0.87 | 0.004 | Reference | | NA | 1.01 | 0.82–1.25 | 0.910 | 1.48 | 1.23–1.78 | <0.001 | 2.08 | 1.57–2.76 | <0.001 | <0.001 |  |
| **Vital signs at mAFP implantation** |  |  |  |  |  |  |  |  |  |  |  |  |  |  |  |  |  |
| Mean blood pressure ≥70 mmHg | 0.66 | 0.44–0.99 | 0.043 | Reference | | NA | 0.99 | 0.76–1.28 | 0.922 | 1.29 | 1.02–1.64 | 0.036 | 2.24 | 1.62–3.08 | <0.001 | <0.001 | 0.775 |
| Mean blood pressure <70 mmHg | 0.73 | 0.55–0.99 | 0.041 | Reference | | NA | 1.05 | 0.86–1.28 | 0.653 | 1.43 | 1.20–1.70 | <0.001 | 1.88 | 1.48–2.38 | <0.001 | <0.001 |  |
| Heart rate ≥90 bpm | 0.71 | 0.50–1.00 | 0.051 | Reference | | NA | 1.07 | 0.85–1.35 | 0.562 | 1.40 | 1.15–1.71 | <0.001 | 1.92 | 1.46–2.52 | <0.001 | <0.001 | 0.871 |
| Heart rate <90 bpm | 0.72 | 0.51–1.00 | 0.051 | Reference | | NA | 0.99 | 0.80–1.24 | 0.959 | 1.31 | 1.07–1.60 | 0.008 | 2.03 | 1.56–2.64 | <0.001 | <0.001 |  |
| **Laboratory values at mAFP implantation** |  |  |  |  |  |  |  |  |  |  |  |  |  |  |  |  |  |
| Lactate ≥8 mmol/L | 0.58 | 0.38–0.87 | 0.008 | Reference | | NA | 1.07 | 0.84–1.36 | 0.602 | 1.63 | 1.32–2.01 | <0.001 | 2.27 | 1.74–2.96 | <0.001 | <0.001 | 0.097 |
| Lactate <8 mmol/L | 0.74 | 0.55–1.00 | 0.049 | Reference | | NA | 1.05 | 0.86–1.30 | 0.617 | 1.25 | 1.03–1.51 | 0.022 | 1.70 | 1.28–2.24 | <0.001 | <0.001 |  |
| Lactate dehydrogenase ≥400 IU/L | 0.64 | 0.46–0.90 | 0.010 | Reference | | NA | 1.03 | 0.84–1.27 | 0.759 | 1.33 | 1.11–1.60 | 0.002 | 1.98 | 1.55–2.54 | <0.001 | <0.001 | 0.903 |
| Lactate dehydrogenase <400 IU/L | 0.80 | 0.57–1.13 | 0.202 | Reference | | NA | 0.98 | 0.77–1.24 | 0.846 | 1.34 | 1.08–1.66 | 0.008 | 2.00 | 1.49–2.70 | <0.001 | <0.001 |  |
| Total bilirubin ≥1.0 mg/dL | 0.71 | 0.48–1.05 | 0.090 | Reference | | NA | 0.96 | 0.73–1.25 | 0.751 | 1.23 | 0.96–1.58 | 0.097 | 1.62 | 1.17–2.26 | 0.004 | <0.001 | 0.145 |
| Total bilirubin <1.0 mg/dL | 0.67 | 0.50–0.91 | 0.011 | Reference | | NA | 1.04 | 0.85–1.27 | 0.690 | 1.45 | 1.22–1.72 | <0.001 | 2.26 | 1.80–2.85 | <0.001 | <0.001 |  |
| Creatinine ≥1.0 mg/dL | 0.72 | 0.56–0.94 | 0.017 | Reference | | NA | 0.96 | 0.81–1.14 | 0.626 | 1.23 | 1.06–1.44 | 0.008 | 1.70 | 1.39–2.09 | <0.001 | <0.001 | 0.165 |
| Creatinine <1.0 mg/dL | 0.72 | 0.41–1.27 | 0.260 | Reference | | NA | 1.36 | 0.93–1.99 | 0.117 | 1.66 | 1.17–2.34 | 0.004 | 3.34 | 2.05–5.43 | <0.001 | <0.001 |  |
| Albumin ≥3.0 g/dL | 0.62 | 0.45–0.86 | 0.004 | Reference | | NA | 1.04 | 0.86–1.26 | 0.691 | 1.30 | 1.09–1.55 | 0.003 | 1.92 | 1.52–2.43 | <0.001 | <0.001 | 0.694 |
| Albumin <3.0 g/dL | 0.79 | 0.55–1.14 | 0.202 | Reference | | NA | 0.99 | 0.75–1.31 | 0.943 | 1.51 | 1.20–1.91 | <0.001 | 2.14 | 1.55–2.94 | <0.001 | <0.001 |  |
| PCI under mAFP | 0.72 | 0.50–1.06 | 0.096 | Reference | | NA | 1.11 | 0.88–1.41 | 0.374 | 1.33 | 1.06–1.67 | 0.013 | 1.93 | 1.38–2.70 | <0.001 | <0.001 | 0.567 |
| No PCI under mAFP | 0.68 | 0.50–0.92 | 0.013 | Reference | | NA | 0.98 | 0.79–1.22 | 0.867 | 1.42 | 1.19–1.70 | <0.001 | 2.06 | 1.64–2.59 | <0.001 | <0.001 |  |
| Upgrade to Impella 5.0 or 5.5 | 0.40 | 0.08–1.92 | 0.251 | Reference | | NA | 0.75 | 0.27–2.09 | 0.579 | 1.78 | 0.79–4.00 | 0.164 | 0.88 | 0.22–3.54 | 0.855 | 0.115 | 0.317 |
| No upgrade to Impella 5.0 or 5.5 | 0.72 | 0.56–0.92 | 0.007 | Reference | | NA | 1.05 | 0.90–1.23 | 0.543 | 1.36 | 1.18–1.56 | <0.001 | 2.04 | 1.69–2.47 | <0.001 | <0.001 |  |
| Insertion site |  |  |  |  | |  |  |  |  |  |  |  |  |  |  |  |  |
| Femoral | 0.71 | 0.55–0.90 | 0.005 | Reference | | NA | 1.03 | 0.88–1.21 | 0.724 | 1.34 | 1.16–1.55 | <0.001 | 1.96 | 1.61–2.38 | <0.001 | <0.001 | 0.768 |
| Other than femoral | 0.84 | 0.25–2.84 | 0.776 | Reference | | NA | 1.02 | 0.40–2.60 | 0.969 | 1.98 | 1.00–3.95 | 0.051 | 3.43 | 1.60–7.34 | 0.002 | <0.001 |  |

aHR = adjusted hazard ratio; AMI = acute myocardial infarction; BMI = body mass index; CI = confidence interval; ECMO = extracorporeal membrane oxygenation; IABP = intra-aortic balloon pump; LVEF = left ventricular ejection fraction; mAFP = microaxial flow pump; NA = not applicable; PCI = percutaneous coronary intervention; STEMI = ST-segment elevation myocardial infarction; TIA = transient ischemic attack.

**Supplemental Table 4. Mortality across BMI categories according to mAFP upgrade status.**

|  | **Overall** | **Underweight** | **Normal weight** | **Overweight** | **Obesity** | **Severe obesity** | **P value*** |
| --- | --- | --- | --- | --- | --- | --- | --- |
| No upgrade from Impella 2.5 or CP | 1,216/3,370 (36.1%) | 78/315 (24.8%) | 421/1,300 (32.4%) | 229/655 (35.0%) | 346/848 (40.8%) | 142/252 (56.3%) | <0.001 |
| Upgrade to Impella 5.0 or 5.5 | 42/138 (30.4%) | 2/11 (18.2%) | 10/39 (25.6%) | 7/27 (25.9%) | 21/49 (42.9%) | 2/12 (16.7%) | 0.191 |
| *P value†* | 0.206 | 1.000 | 0.475 | 0.447 | 0.893 | 0.014 |  |

Data are presented as n/N (percentage). BMI = body mass index; mAFP = microaxial flow pump.

* P value for the comparison across BMI categories.

† P value for no upgrade from Impella 2.5 or CP vs. upgrade to Impella 5.0 or 5.5.
